# Social determinants of participant recruitment and retention in a prospective cohort study of pediatric mild traumatic brain injury

**DOI:** 10.3389/fneur.2022.961024

**Published:** 2022-09-13

**Authors:** Jordee M. Wells, Jean-Michel Galarneau, Nori M. Minich, Daniel M. Cohen, Kameron Clinton, H. Gerry Taylor, Erin D. Bigler, Ann Bacevice, Leslie K. Mihalov, Barbara A. Bangert, Nicholas A. Zumberge, Keith Owen Yeates

**Affiliations:** ^1^Department of Pediatrics, Abigail Wexner Research Institute at Nationwide Children's Hospital, The Ohio State University, Columbus, OH, United States; ^2^Department of Psychology, Alberta Children's Hospital Research Institute, Hotchkiss Brain Institute, University of Calgary, Calgary, AB, Canada; ^3^Department of Pediatrics, Rainbow Babies and Children's Hospital, Case Western Reserve University, Cleveland, OH, United States; ^4^Department of Psychology and Neuroscience Center, Brigham Young University, Provo, UT, United States; ^5^Departments of Radiology and Neurosurgery, University Hospitals Health System, Cleveland, OH, United States; ^6^Department of Radiology, Nationwide Children's Hospital, Columbus, OH, United States

**Keywords:** attrition, concussion, mild traumatic brain injuries (mTBI), pediatric traumatic brain injury, social determinants

## Abstract

**Background:**

Prior studies have shown poor recruitment and retention of minoritized groups in clinical trials.

**Objective:**

To examine several social determinants as predictors of consent to participate and retention as part of a prospective, longitudinal cohort study of children 8–16 with either mild traumatic brain injury (mild TBI) or orthopedic injury (OI).

**Methods:**

Children and families were recruited during acute visits to emergency departments (ED) in two large children's hospitals in the midwestern United States for a prospective, longitudinal cohort study of children 8–16 with either mild TBI or OI.

**Results:**

A total of 588 (mild TBI = 307; OI = 281) eligible children were approached in the ED and 315 (mild TBI = 195; OI = 120) were consented. Children who consented did not differ significantly from those who did not consent in sex or age. Consent rates were higher among Black (60.9%) and multi-racial (76.3%) children than white (45.3%) children. Among the 315 children who consented, 217 returned for a post-acute assessment (mild TBI = 143; OI = 74), a retention rate of 68.9%. Participants who were multi-racial (96.6%) or white (79.8%) were more likely to return for the post-acute visit than those who were Black (54.3%).

**Conclusions:**

Racial differences exist in both recruitment and retention of participants in a prospective, longitudinal cohort of children with mild TBI or OI. Further work is needed to understand these differences to ensure equitable participation of minoritized groups in brain injury research.

## Introduction

Minoritized groups have historically been and continue to be underrepresented in clinical trials (1). While Blacks and Hispanics represented 12 and 16% of the US population in a 2011 report, respectively, only 5 and 1% of trial participants were Black or Hispanic, respectively (2). Lack of diversity in clinical trials is equally a moral and scientific issue that demands a call to action (3). Both homogenous recruitment practices and attrition become a major threat to internal, external, and statistical validity of intervention studies (4, 5). Patient populations within medical research need to reflect real world patterns of representation to address health disparities, advance science, and improve patient outcomes (6).

Pediatric traumatic brain injury (TBI) is a major global public health concern that affects millions of children annually (7). Recent calls to address health disparities in pediatric trauma care underscore the imperative to examine factors related to participant recruitment and retention in research on pediatric TBI (8, 9). Research has clearly shown that Black children demonstrate increased morbidity, mortality, and functional deficit after TBI compared to equivalently injured White children (10–12). The large majority of all TBI sustained by children (about 85–90%) are classified as mild, including concussion (7, 13). Outcomes, including racial and ethnic disparities, from pediatric mild TBI can be highly heterogeneous and difficult to predict from one child to the next (14–16). Improved diversity in clinical trials is essential to refining our understanding of pediatric brain injury in historically excluded groups.

Increasing the participation of minoritized groups in research on brain injury depends on an understanding of the factors associated with participant *recruitment* and *retention*. Systematic reviews have supported various strategies for participant recruitment and retention (17). Yet, the impact of race and other social determinants on recruitment and retention within pediatric mild TBI studies is unknown. Therefore, our study examines several social determinants (e.g., race, socioeconomic status) as predictors of consent to participate and retention as part of a prospective, longitudinal cohort study of children 8–16 with either mild traumatic brain injury (mild TBI) or orthopedic injury (OI).

## Methods

### Study design and procedure

Data were drawn from a large study of pediatric mild TBI that involved a prospective, concurrent cohort design with longitudinal follow-up. Children with mild TBI or OI between the ages of 8 and 16.99 years at the time of injury were recruited during emergency department (ED) visits within 24 h post-injury at Nationwide Children's Hospital (NCH) in Columbus, OH and Rainbow Babies and Children's Hospital (RBCH) in Cleveland, OH. Recruitment occurred over a period of 46 months, from March 2014 to December 2017, to accrue the desired sample size. The study was approved by institutional review boards at NCH and RBCH.

Information regarding the child's injury, sex, age, self-reported race, self-reported ethnicity, and acute clinical presentation was obtained during the ED visit. Parents also provided the address of the family residence, which was used to obtain 2010 Census tract measures of median family income and percentage minority status to assess social determinants at a community level. Basic demographic information (type of injury, sex, age, race, census tract measures) was retained in de-identified format for all eligible children. Informed parental consent and child assent were obtained in writing before formal study participation. Enrolled children were to return three post-injury assessments: post-acute (target within 2 weeks; range = 3–18 days, Mdn = 11.00, M = 10.31, SD = 2.81) and at 3- and 6-months post-injury. The post-acute assessment included magnetic resonance imaging (MRI) of the brain.

### Participants and recruitment

Children with mild TBI were eligible if they experienced a blunt head trauma that resulted in at least one of the following: (a) an observed loss of consciousness for <30 min, (b) Glasgow Coma Scale (GCS) of 13 or 14, or (c) at least two acute signs of symptoms of concussion as noted on standard case report forms (e.g., post-traumatic amnesia, focal neurological deficits, skull fracture, vomiting, headache, dizziness, or other mental status changes). Children with mild TBI were eligible to participate even if they had other minor injuries.

Children with OI were eligible if they experienced an upper or lower extremity fracture with an Abbreviated Injury Scale (AIS) score of 4 or less (18). Children with OI were excluded from the study if they showed any evidence of head trauma or any acute signs or symptoms of concussion.

Both groups were subject to the following exclusion criteria: (a) any other severe injury as defined by an AIS score >4, (b) any associated injury likely to interfere with neuropsychological testing, (c) hypoxia, hypotension, or shock during or following the injury, (d) alcohol or drug ingestion involved with the injury, (e) history of previous TBI or severe psychiatric disorder requiring hospitalization, (f) premorbid neurological disorder or intellectual disability, (g) injury resulting from abuse or assault, or (h) any contraindication to MRI. Children who were administered analgesic medication, including narcotics, were not excluded from either group. Additionally, children with a history of learning or attention problems were not excluded because they are at increased risk for sustaining traumatic injuries (19).

### Demographic information

Additional data regarding social determinants were collected at the post-acute visit, including family income, maternal years of education, marital status, and travel distance to the hospital. We collected data at both the census tract level (median family income and percent minority population) and the individual level (maternal education, marital status, distance traveled). The FFIEC Geocoding/Mapping System (https://geomap.ffiec.gov/FFIECGeocMap/GeocodeMap1.aspx) was used to obtain 2010 Census tract data. The 2010 Census tract data was used because it was the first census that preceded the study period. Race and ethnicity data were used in analyses as a social construct due to their association with differences in ED care-seeking behavior and injury rates (20–22).

### Statistical analysis

Demographic data were analyzed using analysis of variance (ANOVA) for continuous variables (e.g., age, SES) and chi-square techniques for categorical variables (e.g., sex). Bivariate analyses evaluated the unadjusted odds of consent, return for post-acute assessment if consented, and return for 3- and 6- month visits if attending the post-acute assessment, as a function of injury characteristics, demographics, and social factors. Multivariable analyses evaluated the adjusted odds of consent and retention at the census and individual levels separately because of collinearity among some predictors (e.g., census tract percentage minority status and race). The Wald test was used for multivariable logistic regression analysis. All statistical analyses were performed using Stata software, copyright© 1996-2022 StataCorp LLC.

## Results

Sample characteristics and demographic data are presented in Table 1. A total of 588 (mild TBI = 307; OI = 281) eligible children were approached in the ED about participating in the study. Of those children, 315 (mild TBI = 195; OI = 120) were consented and completed the initial assessment in the ED. Children who consented did not differ significantly from those who did not consent in sex or age in bivariate or multivariable analyses (Table 2). A higher proportion of participants consented in the mild TBI group (63.5%) than in the OI group (42.7%). Overall consent rates were higher among Black (60.9%) and multi-racial (76.3%) children than white (45.3%) children. In bivariate analyses, those who consented also lived in census tracts with significantly lower median family incomes, but this was not so in the multivariable model. Consent was significantly related to percent of minorities in children's census tracts in both the bivariate and multivariable analyses, with higher rates of consent in census tracts with higher percent minorities.

**Table 1:** Characteristics of eligible children (*N* = 588).

	***N***	**Mean**	**Median**	**SD**	**Min**	**p25**	**p75**	**Max**
**Age**	583	11.96	12.00	2.44	8.00	10.00	14.00	16.00
**Median family income (Census) per 1000$**	579	60.59	53.61	34.06	6.43	35.44	79.35	247
**Percent minority (Census)**	579	42.59	30.94	34.38	1.23	11.80	76.75	99.75
**Total ISS**	581	2.61	2.00	1.74	1.00	1.00	4.00	13.00
	**N**	**%**						
**Consent**								
Refused	273	46.43						
Consented	315	53.57						
**Post acute visit**								
Refusal or seen at ED only	371	63.10						
Seen for post acute visit	217	36.90						
**Group**								
OI	281	47.79						
mTBI	307	52.21						
**Site**								
Columbus	390	66.33						
Cleveland	198	33.67						
**Sex**								
Female	207	35.38						
Male	378	64.62						
**Race**								
White	285	48.47						
Black	248	42.18						
Asian	5	0.85						
Pacific islander	1	0.17						
Am Ind/Alaskan	2	0.34						
Other	2	0.34						
Multi-racial	38	6.46						
Unknown	7	1.19						

**Table 2:** Multivariable model–odds of eligible children consenting.

	**Census factors**					**Individual factors**				
	**95% CI**					**95% CI**				
	**OR**	**SE**	**Lower**	**Upper**	***P* value**	**OR**	**SE**	**Lower**	**Upper**	***P* value**
**Age**	0.978	0.036	0.911	1.051	0.544	0.977	0.036	0.910	1.050	0.529
**Sex**										
Female	rc					rc				
Male	0.994	0.184	0.691	1.429	0.973	0.954	0.177	0.663	1.373	0.801
**Group**										
OI	rc					rc				
mTBI	2.630	0.506	1.804	3.834	<0.001	2.562	0.491	1.759	3.731	<0.001
**Site**										
Columbus	rc					rc				
Cleveland	1.276	0.279	0.831	1.959	0.265	1.666	0.351	1.102	2.517	0.015
**Total ISS**	1.013	0.055	0.910	1.127	0.818	1.006	0.055	0.905	1.119	0.909
**Median family income (Census) per 1,000$**	1.000	0.003	0.993	1.006	0.937	0.996	0.003	0.990	1.002	0.190
**Percent minority (Census)**	1.013	0.004	1.006	1.021	0.001					
**Race**										0.004^†^
White	rc					rc				
Black						1.519	0.360	0.954	2.416	0.078
Other						3.188	1.155	1.567	6.487	0.001
**Constant**	0.510	0.292	0.166	1.564	0.239	0.833	0.447	0.291	2.383	0.733
**r**^**2**^ **(pseudo)**	0.074					0.074				
**Correctly classified (%)**	64.46					63.94				
**Sensitivity (%)**	45.56					49.42				
**Specificity (%)**	80.00					75.87				
**AUC**	0.683					0.683				

*^†^*Overall test using Wald test; rc, reference category.

N = 574.

Among the 315 children who consented, 217 returned for a post-acute assessment (mild TBI = 143; OI = 74), a retention rate of 68.9% (Table 3). In the multivariable model, children assessed in the ED only did not differ from those who returned for the post-acute assessment in sex, age, injury type, study site, overall injury severity, census tract median family income, or distance to hospital (Table 4). Participants who were multi-racial (96.6%) or white (79.8%) were more likely to return for the post-acute visit than those who were Black (54.3%). Children who returned for the post-acute assessment also resided in census tracts with a lower percentage of minorities than those who were seen only in the ED. Among children completing the post-acute assessment children who returned for the follow up visit at 3- and/or 6-month visits did not differ significantly from those who did not return in age, sex, race, type of injury, distance to hospital, maternal education, or marital status (Table 5), but did reside in census tracts with lower median family income in the multivariable model.

**Table 3:** Characteristics of participants who consented (*N* = 315).

	***N***	**Mean**	**Median**	**SD**	**Min**	**p25**	**p75**	**Max**
**Age**	315	11.95	12.00	2.43	8.00	10.00	14.00	16.00
**Median family income (Census) per 1,000$**	315	56.58	50.89	33.06	6.43	32.50	76.10	244.33
**Percent minority (Census)**	315	49.36	43.30	35.23	2.00	15.48	86.28	99.75
**Total ISS**	315	2.51	2.00	1.78	1.00	1.00	4.00	13.00
**Miles to hospital**	313	8.55	5.53	9.21	0.90	2.96	11.09	70.49
	**N**	**%**						
**Post Acute Visit**								
Seen at ED only	98	31.11						
Seen for post acute visit	217	68.89						
**Group**								
OI	120	38.10						
mTBI	195	61.90						
**Site**								
Columbus	189	60.00						
Cleveland	126	40.00						
**Sex**								
Female	109	34.60						
Male	206	65.40						
**Race**								
White	129	40.95						
Black	151	47.94						
Asian	2	0.63						
Pacific islander	1	0.32						
Am Ind/Alaskan	2	0.63						
Other	1	0.32						
Multi-racial	29	9.21						

**Table 4:** Multivariable model–odds of consented participants returning for post-acute visit.

	**Census factors**					**Individual factors**				
	**95% CI**					**95% CI**				
	**OR**	**SE**	**Lower**	**Upper**	***P* value**	**OR**	**SE**	**Lower**	**Upper**	***P* value**
**Age**	0.994	0.053	0.895	1.103	0.909	1.004	0.055	0.901	1.118	0.942
**Sex**										
Female	rc					rc				
Male	1.167	0.316	0.687	1.983	0.568	1.341	0.375	0.775	2.321	0.295
**Group**										
OI	rc					rc				
mTBI	1.271	0.359	0.730	2.213	0.396	1.343	0.389	0.761	2.370	0.310
**Site**										
Columbus	rc					rc				
Cleveland	0.645	0.190	0.361	1.150	0.137	0.729	0.212	0.413	1.288	0.276
**Total ISS**	0.896	0.067	0.775	1.037	0.140	0.892	0.068	0.768	1.037	0.137
**Median family income (Census) per 1,000$**	1.001	0.006	0.990	1.012	0.868	1.001	0.005	0.991	1.011	0.850
**Percent minority (Census)**	0.986	0.007	0.973	0.999	0.029					
**Miles to hospital**	0.976	0.017	0.943	1.011	0.173	0.980	0.016	0.950	1.012	0.220
**Race**										<0.001^†^
White						rc				
Black						0.283	0.116	0.127	0.632	0.002
Other						2.615	1.761	0.699	9.787	0.154
**Constant**	7.366	7.132	1.104	49.129	0.039	4.612	3.891	0.883	24.102	0.070
**r**^**2**^ **(pseudo)**	0.067					0.108				
**Correctly classified (%)**	69.01					72.20				
**Sensitivity (%)**	19.39					30.61				
**Specificity (%)**	91.63					91.16				
**AUC**	0.677					0.715				

*^†^*Overall test using Wald test; rc, reference category.

N = 313.

**Table 5:** Multivariable model–odds of returning for a follow-up visit after completing post-acute visit.

	**Census factors**					**Individual factors**				
	**95% CI**					**95% CI**				
	**OR**	**SE**	**Lower**	**Upper**	***P* value**	**OR**	**SE**	**Lower**	**Upper**	***P* value**
**Age**	1.040	0.071	0.909	1.190	0.565	1.040	0.071	0.909	1.189	0.569
**Sex**										
Female	rc					rc				
Male	1.589	0.570	0.787	3.209	0.196	1.584	0.572	0.781	3.213	0.203
**Group**										
OI	rc					rc				
mTBI	0.905	0.359	0.416	1.970	0.802	0.822	0.334	0.371	1.822	0.628
**Site**										
Columbus	rc					rc				
Cleveland	0.607	0.248	0.272	1.352	0.222	0.644	0.260	0.292	1.422	0.276
**Total ISS**	0.926	0.105	0.741	1.157	0.497	0.918	0.106	0.733	1.151	0.459
**Median family income (Census) per 1,000$**	1.018	0.009	1.001	1.034	0.039					
**Percent minority (Census)**	1.008	0.009	0.991	1.026	0.347					
**Miles to hospital**	0.981	0.025	0.933	1.032	0.455	0.965	0.023	0.922	1.010	0.126
**Maternal years education**						1.062	0.086	0.905	1.245	0.461
**Race**										0.602^†^
White						rc				
Black						0.781	0.432	0.265	2.307	0.655
Other						0.583	0.313	0.203	1.668	0.314
**Marital status**										0.807^†^
Married/living with someone						rc				
Never married						0.739	0.344	0.297	1.839	0.515
Widowed/separated/divorced						0.938	0.449	0.367	2.396	0.893
**Constant**	0.807	1.053	0.063	10.419	0.870	2.314	3.537	0.116	46.301	0.583
**r**^**2**^ **(pseudo)**	0.044					0.331				
**Correctly classified (%)**	79.05					78.10				
**Sensitivity (%)**	2.22					0.00				
**Specificity (%)**	100.00					99.39				
**AUC**	0.650					0.624				

*^†^*Overall test using Wald test; rc, reference category.

N = 210.

## Discussion

The current study used data from a multisite, prospective study with longitudinal follow up of children with mild TBI or mild OI to investigate social determinants as predictors of consent to participate and retention in clinical research. Among ED visits for mild TBI and OI, Black and multi-racial children were at least 15% more likely to consent to enrollment in the prospective study compared to White children. However, White and multi-racial children were 30% more likely to return for post-acute visit compared to Black children. In addition, participants in the mild TBI group were more likely to consent to participate compared to participants in the OI group, but the groups did not differ in retention. These findings are consistent with prior data and pose important questions about how clinical diagnosis and social determinants, especially race, affect participant recruitment into clinical research.

Race was the strongest predictor of return for the post-acute visit, whether coded at the census tract or individual level. Hispanic participants were included but underrepresented in our sample, precluding analysis of ethnicity separately from race. White and multiracial participants were more likely to return for the post-acute visit than Black participants. The post-acute visit was time intensive and involved advanced MRI imaging, which may have contributed to a higher percentage of White and multiracial participants returning. Pediatric imaging overuse has been repeatedly shown in non-Hispanic White populations (23). Furthermore, in cases of non-severe head trauma, greater levels of parental anxiety may be apparent in non-Hispanic White populations, and associated with increased requests for advanced imaging (24). The promise of an MRI during the post-acute visit may have been perceived as added value and incentive to White and multiracial participants compared to Black participants.

Race, education, injury type, and most other predictors appeared to have little influence on return for follow up visits at 3- and 6- months if a participant came to the post-acute visit. This suggests that retention at the post-acute visit is the most important step to minimizing the impact of social determinants, as attrition was relatively low among patients who participated in post-acute visits and was not as strongly related to social determinants. Missing first assessment appointments have previously been associated with higher attrition risks (25). Prior research also has suggested that non-response was most common in minoritized groups. Thus, examining social determinants as they relate to non-random participation and attrition is vital to making research relevant to the broader population (26). As longitudinal studies remain incredibly important to examining development in children with medical disorders or injuries, developing strategies to maintain diversity within research participants must be prioritized (17).

Our study provided a relatively unique opportunity to examine several social determinants as predictors of both consent to participate and retention as part of a prospective, longitudinal cohort study. Achieving and retaining a diverse group of participants within clinical trials has been a significant challenge reported in the literature (27). Barriers to recruitment and retention have included age, sex, socioeconomic factors, cultural factors, and practical challenges, including time and travel commitments (28, 29). A socio-ecological model has been suggested as a platform to achieve and maintain high recruitment and retention rates within minoritized groups (30). Race is a strong predictor and should be prioritized with other determinants in clinical trial design as it relates to participant recruitment and retention. We should acknowledge that the role of race is likely secondary to its interactions with multiple social determinants of health. Indeed, race is a complex social construct and a proxy for multiple potential barriers to recruitment and retention that warrants further research.

Pre-emptively employing mitigation strategies as a means to increase participant retention remains important. Our study used multiple strategies to increase retention, including transportation support, accommodating schedules for both evenings and weekends, and allowing non-guardians to accompany the child for follow up appointments. Previous studies have employed community engagement strategies to effectually bring research to neighborhoods and to the home, and prioritized recruiting research personnel who shared racial and ethnic identities with those of participants (31, 32). Further research is required to determine which mitigation strategies will help to achieve and maintain optimal participant retention.

The findings of our study must be interpreted within the context of its limitations. Based on the longitudinal study design and schedule for data collection, we had progressively more data available regarding participants that consented and returned for follow up compared to those who did not consent or did not return for the post-acute visit. We were limited in the amount and type of data that we could collect on participant who did not consent to participate and could not examine characteristics including employment or access to transportation. The study was not originally designed or powered to examine social determinants of recruitment and retention; as a secondary analysis, our study may have benefited from a larger sample size to detect some differences between groups with respect to recruitment and retention. Finally, while race was our strongest predictor, we had some missing data that limited our ability to assign the characteristic to each participant and were unable to analyze Hispanic or Latino ethnicity separately due to small sample size. Therefore, we acknowledge that a small percentage of our participants within the White group included minoritized individuals that identified as Hispanic or Latino.

## Conclusion

Racial differences are apparent in both recruitment and retention of participants in a prospective, longitudinal cohort of children with mild TBI or OI. Black and multiracial participants may be more likely to consent to enrollment in ED-based prospective trials for mild TBI and OI, while White and multiracial participants are more likely to engage in longitudinal follow up. The perceived added value of longitudinal follow up to participants may influence decisions to return for post-acute assessments. Our findings provide important insights into differences in recruitment and participation among minoritized groups in childhood brain injury research.

## Data availability statement

The raw data supporting the conclusions of this article will be made available by the authors, without undue reservation.

## Ethics statement

The studies involving human participants were reviewed and approved by Nationwide Children's Hospital Internal Review Board and Rainbow Babies and Children's Hospital Internal Review Board. Written informed consent to participate in this study was provided by the participants' legal guardian/next of kin.

## Author contributions

JW conceptualized and designed the study, performed data analyses, drafted the initial manuscript, and reviewed and revised the manuscript. DC and KY aided in the conceptualization and design of the study, reviewed data analyses, and reviewed and revised the manuscript. J-MG and NM completed and supervised data analyses and reviewed and revised the manuscript. KC reviewed data analyses, supported the drafting of the initial manuscript, and reviewed and revised the manuscript. All authors approved the final manuscript as submitted and agree to be accountable for all aspects of the work.

## Funding

This work was supported by the National Institutes of Health grant R01HD076885.

## Conflict of interest

The authors declare that the research was conducted in the absence of any commercial or financial relationships that could be construed as a potential conflict of interest.

## Publisher's note

All claims expressed in this article are solely those of the authors and do not necessarily represent those of their affiliated organizations, or those of the publisher, the editors and the reviewers. Any product that may be evaluated in this article, or claim that may be made by its manufacturer, is not guaranteed or endorsed by the publisher.
