# Supplementary material for: Social determinants of participant recruitment and retention in a prospective cohort study of pediatric mild traumatic brain injury
Source: Front Neurol. 2022 Sep 13;13:961024. doi: 10.3389/fneur.2022.961024 (PMC9513452; doi:10.3389/fneur.2022.961024)
Supplement: Supplementary file 1 [file Table_1.DOCX]

Supplementary Material

# Supplementary Data

| **Supplementary Table 1: Characteristics of Participants Who Returned for Post-Acute (N=217)** | | | | | | | | |
| --- | --- | --- | --- | --- | --- | --- | --- | --- |
|  | **N** | **Mean** | **Median** | **SD** | **min** | **p25** | **p75** | **max** |
| **Age** | 217 | 11.96 | 12.00 | 2.53 | 8.00 | 10.00 | 14.00 | 16.00 |
| **Median family income (Census) per 1000$** | 217 | 60.05 | 53.45 | 34.18 | 7.33 | 35.07 | 79.29 | 244.33 |
| **Percent minority (Census)** | 217 | 43.94 | 31.66 | 34.47 | 2.00 | 13.10 | 77.00 | 99.75 |
| **Total ISS** | 217 | 2.37 | 2.00 | 1.58 | 1.00 | 1.00 | 4.00 | 10.00 |
| **Miles to hospital** | 215 | 9.02 | 6.46 | 8.10 | 0.92 | 3.44 | 12.05 | 42.41 |
| **Maternal years education** | 216 | 13.70 | 13.00 | 2.34 | 9.00 | 12.00 | 16.00 | 18.00 |
|  | **N** | **%** |  |  |  |  |  |  |
| **Last Visit** |  |  |  |  |  |  |  |  |
| 3m and/or 6m | 170 | 78.34 |  |  |  |  |  |  |
| No 3m & No 6m | 47 | 21.66 |  |  |  |  |  |  |
| **Group** |  |  |  |  |  |  |  |  |
| OI | 74 | 34.10 |  |  |  |  |  |  |
| mTBI | 143 | 65.90 |  |  |  |  |  |  |
| **Site** |  |  |  |  |  |  |  |  |
| Columbus | 144 | 66.36 |  |  |  |  |  |  |
| Cleveland | 73 | 33.64 |  |  |  |  |  |  |
| **Sex** |  |  |  |  |  |  |  |  |
| Female | 74 | 34.10 |  |  |  |  |  |  |
| Male | 143 | 65.90 |  |  |  |  |  |  |
| **Race** |  |  |  |  |  |  |  |  |
| White | 103 | 47.47 |  |  |  |  |  |  |
| Black | 82 | 37.79 |  |  |  |  |  |  |
| Asian | 1 | 0.46 |  |  |  |  |  |  |
| Pacific Islander | 1 | 0.46 |  |  |  |  |  |  |
| Am Ind/Alaskan | 2 | 0.92 |  |  |  |  |  |  |
| Multi-Racial | 28 | 12.90 |  |  |  |  |  |  |
| **Marital status** |  |  |  |  |  |  |  |  |
| Married | 93 | 43.06 |  |  |  |  |  |  |
| Widowed | 3 | 1.39 |  |  |  |  |  |  |
| Divorced | 28 | 12.96 |  |  |  |  |  |  |
| Separated | 13 | 6.02 |  |  |  |  |  |  |
| Never Married | 64 | 29.63 |  |  |  |  |  |  |
| Live with someone | 15 | 6.94 |  |  |  |  |  |  |
